# Anti-high mobility group box 1 antibody suppresses local inflammatory reaction and facilitates olfactory nerve recovery following injury

**DOI:** 10.1186/s12974-018-1168-7

**Published:** 2018-04-26

**Authors:** Masayoshi Kobayashi, Kengo Tamari, Mohammed Omar Al Salihi, Kohei Nishida, Kazuhiko Takeuchi

**Affiliations:** Department of Otorhinolaryngology-Head and Neck Surgery, Mie University Graduate School of Medicine, 2-174 Edobashi, Tsu, Mie 514-8507 Japan

**Keywords:** Astrocyte, Cycloheximide, Field potential recording, Head injury, HMGB1, Macrophage, Olfactory bulb, Olfactory nerve, Olfactory marker protein (OMP), Regeneration

## Abstract

**Background:**

Refractory olfactory dysfunction is a common finding in head trauma due to olfactory nerve injury. Anti-inflammatory treatment using steroids is known to contribute to functional recovery of the central and peripheral nervous systems in injury models, while there is a concern that steroids can induce side effects. The present study examines if the inhibition of proinflammatory cytokine, high mobility group box 1 (HMGB1), can facilitate olfactory functional recovery following injury.

**Methods:**

Olfactory nerve transection (NTx) was performed in OMP-tau-lacZ mice to establish injury models. We measured HMGB1 gene expression in the olfactory bulb using semi-quantitative polymerase chain reaction (PCR) assays and examined HMGB1 protein localization in the olfactory bulb using immunohistochemical staining. Anti-HMGB1 antibody was intraperitoneally injected immediately after the NTx and histological assessment of recovery within the olfactory bulb was performed at 5, 14, 42, and 100 days after the drug injection. X-gal staining labeled OMP in the degenerating and regenerating olfactory nerve fibers, and immunohistochemical staining detected the presence of reactive astrocytes and macrophages/microglia. Olfactory function was assessed using both an olfactory avoidance behavioral test and evoked potential recording.

**Results:**

HMGB1 gene and protein were significantly expressed in the olfactory bulb 12 h after NTx. Anti-HMGB1 antibody-injected mice showed significantly smaller areas of injury-associated tissue, fewer astrocytes and macrophages/microglia and an increase in regenerating nerve fibers. Both an olfactory avoidance behavioral test and evoked potential recordings showed improved functional recovery in the anti-HMGB1 antibody-injected mice.

**Conclusions:**

These findings suggest that inhibition of HMGB1 could provide a new therapeutic strategy for the treatment of olfactory dysfunction following head injuries.

## Background

Olfactory dysfunction lowers our quality of life and can be life threatening because of the inability to detect hazardous events such as fire, gas leak, and spoiled food intake [1, 2]. Head trauma is one of the major causes of olfactory dysfunction due to overextension, distortion, and tearing of the olfactory nerves and contusions of the olfactory bulbs and orbitofrontal regions of the brain [3]. A major problem with traumatic olfactory dysfunction is the poor prognosis for recovery. Although the olfactory system has a remarkable capacity for neural regeneration and recovery after injury, the clinical improvement rate for olfactory dysfunction in patients with head trauma is only 10–38% [4–8] while that with chronic rhinosinusitis and allergic rhinitis is reported to be 68–86% [9–11]. Therefore, development of therapeutic management for olfactory dysfunction is an important clinical issue.

We previously reported using an olfactory nerve injury model in mice that anti-inflammatory treatment with steroids, anti-interleukin-6 (IL-6) receptor antibody, or tumor necrosis factor (TNF)-α blocker, during the acute phase of injury is effective in suppressing the inflammatory reaction and local glial scar formation and improves recovery outcomes after olfactory nerve transection (NTx) [12–14]. In clinical practice, however, these drugs are not typically used for the treatment of head injury patients since several studies reported that steroids do not have a significant efficacy on morbidity and mortality in patients with severe head injury, and there are concerns that steroids may cause serious side effects such as hypertension, hyperglycemia, infection, bone necrosis, and psychosis [15–17]. Although there are fewer concerns about anti-IL-6 receptor antibody and TNF-α blocker use, their administration may sometimes induce severe infection due to excessive suppression of the immune system [18, 19].

High mobility group box 1 (HMGB1), which is originally reported as a nuclear DNA-binding protein contributing to maintenance of nucleosome structure and regulation of gene transcription, is known to be widely expressed in immune and other vertebrate cells, including neurons [20]. Currently, HMGB1 is considered to be an important contributor to the inflammatory process. Once released into the extracellular space from damaged cells, it activates an inflammatory response via activation of multiple receptors such as the receptor for advanced glycation end product (RAGE) and toll-like receptor (TLR) 2 and TLR4 [21, 22]. In the central nervous system, previous studies reported that HMGB1 is upregulated in a spinal cord injury mouse model and that it is associated with neuronal cell apoptosis, suggesting that it can be a therapeutic target for spinal cord injury [23, 24]. In addition, a recent study reported that blockade of HMGB1 using anti-HMGB1 antibody reduces acute brain edema after traumatic brain injury through inhibition of inflammatory responses [25].

The present study was designed to investigate if therapeutic intervention using anti-HMGB1 antibody is effective in improving recovery outcomes in the olfactory system following injury in mice. In this study, we first demonstrated an increase in levels of HMGB1 in the injured olfactory system using a polymerase chain reaction (PCR) assay and immunohistochemical staining techniques, which would be a target of the anti-HMGB1 antibody. Subsequently, we used histological techniques to examine the efficacy of the anti-HMGB1 antibody on recovery outcome by measuring the degree of degeneration and regeneration of olfactory nerve fibers and the amount of injury-associated tissue (glial scar), reactive astrocytes, and macrophages/microglia. We also administered an olfactory function test using avoidance conditioning behavior to odorants as well as electrophysiological recording of field potential responses to electrical stimulation of the olfactory mucosa to determine if morphological recovery parallels functional recovery in the olfactory system following therapeutic intervention.

## Methods

### Experimental animals

This study was performed using transgenic mice (OMP-tau-lacZ mice) obtained from the Jackson Laboratory (Bar Harbor, ME, USA), whose strain is derived from C57BL/6 mice. In this strain, the gene sequence encoding the olfactory marker protein (OMP) has been replaced with a tau-lacZ reporter gene [26]. The OMP is expressed in all mature olfactory neurons [27], and the replacement with tau-lacZ reporter gene enables the visualization of olfactory nerve fibers and their projections to olfactory bulb glomeruli. The advantage of using these mice is that a histological assessment of degenerating and regenerating olfactory nerve fibers can be performed using a standard method for staining and light microscopy. Although the exact function of OMP and effects of lack of OMP has not been revealed yet, many previous studies have shown that OMP-tau-lacZ mice are capable of recovering olfactory function after olfactory nerve injury [13, 14].

### Surgical procedure

**Fig. 1:**
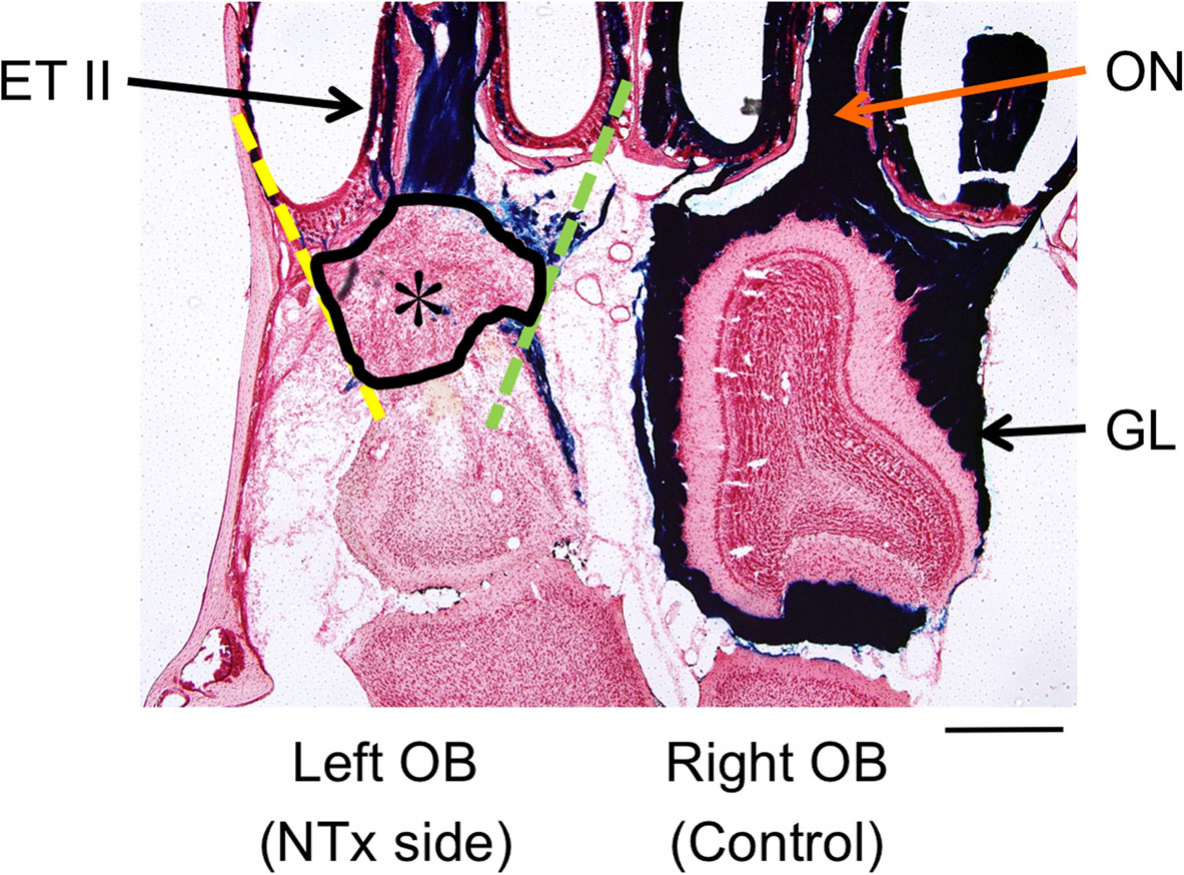
An experimental mouse model of severe olfactory bulb deafferentation injury. A horizontal section through the nasal cavities and olfactory bulbs illustrating differences observed between the lesioned (left) and control (right) sides at 5 days after a nerve transection (NTx) injury. The area of injury-associated tissue (enclosed by a black line, *) was measured and quantified within an area surrounded by the cribriform plate (an anterior margin), anterior edge of the olfactory bulb (a posterior margin), a line connecting posterior end of the nasal septal mucosa and the anteriomedial corner of the olfactory bulb (medial margin, green dotted line) and a line connecting posteriolateral end of the most lateral sinus and the anteriolateral corner of the olfactory bulb (lateral margin, yellow dotted line), using ImageJ software (ver.1.50i, NIH, USA). The olfactory nerves and their projections to the glomeruli are labeled using an X-gal staining method (blue color**)**. GL glomerular layer, OB olfactory bulb, ON olfactory nerve, ET II endoturbinate II. Calibration bar = 500 μm

Both male and female adult mice were used in this study and randomly assigned to experimental groups. Mice were anesthetized with sodium pentobarbital (80 mg/kg, ip). Under sufficient anesthesia, a frontal craniotomy was performed to expose the olfactory bulbs. An olfactory nerve transection procedure (NTx) was performed between the olfactory bulb and cribriform plate using a curved rigid stainless steel blade to generate a severe olfactory nerve injury model [15]. For histological assessments, the NTx procedure was performed only on the left side (injury side) of the animal while the right side (right olfactory bulb and nerves) remained intact and served as an internal histological control (Fig. 1). For other assessments (PCR, olfactory function testing, evoked potentials), a bilateral NTx was performed, cutting the olfactory nerves to the right and left olfactory bulbs, resulting in a complete loss of smell (anosmia). After the NTx procedure was complete, the skin incision was sutured and the animal closely monitored until it was awake and fully recovered from anesthesia. All protocols and surgical procedures for this study were reviewed and approved by the Institutional Animal Care and Use Committee of Mie University.

### PCR

For the PCR assays, mice (*n* = 30) were divided into groups according to the time of sacrifice after the bilateral NTx surgery (1, 3, 6, 12, 24, and 48 h and 5, 10, and 14 days). An additional control group (0 h) was included that did not have any surgery. The olfactory bulbs were removed immediately after sacrifice and the anteroventral part sectioned off and stored at − 80 °C for semi-quantitative PCR HMGB1 assays.

The olfactory bulb tissue samples were placed into Sepasol Super G and homogenized. Total RNA was extracted and purified by DNase treatment according to the manufacturer’s protocols. The reverse transcription reaction was performed using the ReverTraAce (Toyobo, Japan) using 1 μg of total RNA. Variation in the reverse transcription reaction was limited by transcribing all samples simultaneously. PCR reactions were optimized to 94 °C for 2 min, 35 amplification cycles for HMGB1 and 26 cycles for GAPDH at 94 °C for 10 s, 60 °C for 20 s, 72 °C for 40 s, and a final extension of 2 min at 72 °C. Amplified products were resolved on 1.5% agarose gel and visualized by ethidium bromide staining using ITLS software.

Primer Sequences

HMGB1: Primer Bank ID 6754208a1

Forward: GGCGAGCATCCTGGCTTATC

Reverse: GGCTGCTTGTCATCTGCTG

Amplicon size: 86 bp

GAPDH: Primer Bank ID: 126012538c3

Forward: TGGCC TTCCG TGTTC CTAC

Reverse: GAGTT GCTGT TGAAG TCGCA

Amplicon size: 178 bp

### Anti-HMGB1 antibody injection

To investigate the associations among HMGB1, the inflammatory reaction, and nerve regeneration after injury, the anti-HMGB1 antibody (Novus Biologicals, USA) was injected intraperitoneally just after the NTx. To determine if there is a dose-dependent effect of the drug, low (25 μg in 0.5 ml saline) and high (50 μg in 0.5 ml saline) doses of the anti-HMGB1 antibody were used, making them comparable to a dose used in a previous study [25]. We also injected IgG (50 μg in 0.5 ml saline) intraperitoneally in a group of control animals. We collected data from six mice for each of the three treatment groups and each of the four recovery time points (days 5, 14, 42, and 100) for a total of 72 mice (6 mice × 3 doses × 4 recovery points).

### Tissue preparation

For histological assays, mice were anesthetized on the assigned post recovery day with sodium pentobarbital (80 mg/kg, ip) and fixed by intracardiac perfusion using 4% paraformaldehyde in phosphate buffer after a saline rinse. The nasal cavity and anterior portion of the skull were removed *en bloc* and postfixed by immersion in 4% paraformaldehyde for 45 min and then placed in 0.5 M EDTA (ethylenediaminetetraacetic acid) for decalcification for 14 days. The tissue was cryoprotected with 30% sucrose for 2 days, then immersed in embedding compound, quickly frozen in a − 80 °C freezer, and sectioned on a cryostat. Serial horizontal sections through the nasal cavities and olfactory bulbs along the dorsum nasi were cut at 30 μm and mounted on glass slides.

### X-gal staining

Tissue sections were washed at room temperature with buffer A [100 mM phosphate buffer (pH 7.4), 2 mM MgCl_2_, and 5 mM EGTA (ethylene glycol tetraacetic acid)] once for 5 min and then a second time for 25 min. This was followed by two 5-min washes with buffer B [100 mM phosphate buffer (pH 7.4), 2 mM MgCl_2_, 0.01% sodium deoxycholate, and 0.02% Nonidet P40]. The blue X-gal reaction was generated overnight in the dark by exposure to buffer C (buffer B, with 5 mM potassium ferricyanide, 5 mM potassium ferrocyanide, and 1 mg/ml of X-gal). The X-gal reaction was stopped by two 5-min washes in phosphate buffer.

### Measurement of injury-associated tissue and nerve recovery

After confirming the appearance of the blue X-gal reaction, tissue sections were counterstained with a 1% Neutral Red solution. Sections were examined and digitized using CCD photomicroscopy. Areas of injury-associated tissue, including inflammatory cells and glial scar tissue, were identified along with blue (X-gal) labeled olfactory nerve endings within the glomerular layer of the olfactory bulb (Fig. 1). The area of injury-associated tissue was outlined on digital images of tissue sections and quantified using ImageJ (ver.1.50i, National Institute of Health [NIH], USA) software. For the measurement of injury-associated tissue, we targeted an area that was surrounded by the following four margins: the cribriform plate as an anterior margin, anterior edge of the olfactory bulb as a posterior margin, a line connecting the posterior end of the nasal septal mucosa and the anteriomedial corner of the olfactory bulb as a medial margin, and a line connecting the posteriolateral end of the most lateral sinus and the anteriolateral corner of the olfactory bulb as a lateral margin. Since this targeted area is where the olfactory nerve fibers normally run from the sinonasal mucosa to the olfactory bulb before NTx, as seen in the control side, the tissue levels in this area can be associated with the degree of nerve degeneration and regeneration.

The area (mm^2^) of tissue observed between the cribriform plate and olfactory bulb (Fig. 1) was measured in two representative horizontal sections (sections A and B) from each animal and averaged. Section A was selected to represent the dorsal level. At this particular level, a large olfactory nerve bundle is observed passing from endoturbinate II through the cribriform plate to the olfactory bulb (Fig. 1). Section B represented a more ventral level. At this level, endoturbinate III attaches to the cribriform plate. The area measurements from NTx mice at each of the four recovery time points were used to compare mean values for injury-associated tissue. The levels of olfactory nerve degeneration and regeneration were assessed by comparing changes in the amount of blue X-gal staining in the glomerular layer on the left (NTx injury side) to that on the right (control) side. Horizontal olfactory bulb sections (sections A and B) were also used to obtain measurements of (1) the glomerular layer perimeter distance (G-P distance), a continuous line passing through the center of all the glomeruli within the bulb section, and (2) the total length of glomerular segments along the perimeter that were labeled with the blue X-gal stain (G-X-gal distance). The ratio of the X-gal-stained distance (G-X-gal distance) to the total perimeter of the glomerular layer (G-P distance) was obtained for both the NTx injury and control sides. Changes in the blue X-gal nerve staining on the NTx injury-left side were expressed as percentage of the X-gal staining on the intact control side and were used to measure levels of olfactory nerve degeneration and regeneration within the olfactory bulb, as follows:

$$ \%\mathrm{olfactory}\ \mathrm{nerve}\ \mathrm{in}\mathrm{nervation}\ \mathrm{in}\ \mathrm{the}\ \mathrm{glomerular}\ \mathrm{layer}=\frac{\frac{\mathrm{G}\hbox{-} \mathrm{X}\hbox{-} \mathrm{gal}\ \mathrm{distance}\ \mathrm{of}\ \mathrm{NTx}\ \mathrm{side}}{\mathrm{G}\hbox{-} \mathrm{P}\ \mathrm{distance}\ \mathrm{of}\ \mathrm{NTx}\ \mathrm{side}}}{\frac{\mathrm{G}\hbox{-} \mathrm{X}\hbox{-} \mathrm{gal}\ \mathrm{distance}\ \mathrm{of}\ \mathrm{control}\ \mathrm{side}}{\mathrm{G}\hbox{-} \mathrm{P}\ \mathrm{distance}\ \mathrm{of}\ \mathrm{control}\ \mathrm{side}}}\times 100\ \left(\%\right) $$

### Immunohistochemical assessment

Immunohistochemical staining for HMGB1 protein, glial fibrillary acidic protein (GFAP), and cluster of differentiation (CD) 68 glycoprotein was performed on horizontal sections of NTx or control mice. HMGB1 staining was performed at two different time points following left NTx injury, 12- and 24-h points. GFAP and CD68 staining was performed at four different time points following left NTx injury, days 5, 14, 42, and 100. GFAP is constitutively produced by astrocytes. In the reactive glial response to the central nervous system injury, hypertrophic reactive astrocytes increase their expression of GFAP [28]. CD68 staining was used to measure injury-induced inflammatory changes at different time points after NTx injury. CD68 is a lysosomal membrane-associated glycoprotein that is expressed on the surface of histiocytes, cells that are part of the immune system, including macrophages and microglia and play an important role in phagocytic activities.

After washing with phosphate-buffer saline (PBS) for 5 min, sections were processed by immersion for 1-min intervals in a series of alcohol solutions (70, 95, 100, 95, and 70% ethanol). This was followed by three 5-min washes with 0.3% Triton X-100 in PBS. Sections were then incubated with 5% normal goat serum, 1% bovine serum albumin, and 0.5% Triton X-100 in PBS for 30 min and reacted with one of the following primary antibodies: rabbit anti-HMGB1 antibody (Novus Biologicals, USA), rabbit anti-mouse GFAP antibody (1:500, DAKO, USA), and rat anti-mouse CD68 antibody (1:100, AbD serotec, USA). Anti-HMGB1 antibody was visualized using biotinylated goat anti-rabbit IgG (Vector Laboratories, USA) and horseradish peroxidase streptavidin (Vector Laboratories, USA). Diaminobenzidine (DAB) was used as a chromogen. Anti-GFAP and CD68 antibodies were visualized using Cy3-conjugated goat anti-rabbit IgG (1:100, GE, USA) and Alexa Fluor 488-conjugated goat anti-rat IgG (1:100, Invitrogen, USA) under fluorescent microscope, respectively. GFAP- and CD68-positive cells were counted in five different 0.01-mm^2^ sampling areas located in the anterior (injured) region of the olfactory bulb (five areas: the anterior apex area, areas of anteriomedial corner and anteriolateral corner of the olfactory bulb, and fixed midpoint areas between the anterior apex area and anteriomedial and anteriolateral corner areas). The average number of GFAP- and CD68-positive cells/0.01mm^2^ was then calculated for NTx mice at each of the four recovery time points.

### Olfactory function test

To determine if olfactory function recovered after the NTx, a smell detection test using avoidance conditioning behavior to cycloheximide was administered to mice before and after the NTx as reported previously [13, 14]. Cycloheximide has a peculiar odor and unpleasant taste for mice. Normal mice were first deprived of water for 48 h and then trained to avoid cycloheximide solution. Before NTx surgery, mice were conditioned in two or more training sessions, each consisting of 10 trials. In each trial, the mouse was presented with bottles of 0.01% cycloheximide solution and distilled water, one positioned on the left, the other on the right side of a test cage. When the mouse licked the delivery tube of either bottle, the bottles were withdrawn from view and presented again. The left and right positions of the two bottles were shifted according to the Gellermann series (cycloheximide bottle position: right (R)-left (L)-L-R-L-L-R-R-R-L). Mice were considered to have learned the smell of cycloheximide when they chose the distilled water bottle 10 consecutive times out of 10 trials (percent score: 100%) on two consecutive test sessions. After NTx surgery, the test was administered every 7 days until the mouse regained its olfactory function (scored 10 out of 10 correct responses) or exceeded a 100-day cutoff period. Mice that scored 100% at one of the recovery test days were considered to have fully recovered their olfactory function.

### Evoked field potential recording

To confirm that regenerated olfactory receptor cell axons were functionally reconnected to the olfactory bulb after the olfactory NTx, field potentials evoked by electrical stimulation of the olfactory mucosa were measured in the olfactory bulb of mice that completed the cycloheximide olfactory function test as reported previously [13, 14]. For this group of mice, the NTx procedure was bilateral, resulting in a complete loss of olfactory function. On day 100, the animal was anesthetized and the nasal bone removed to expose the olfactory mucosa, and a second craniotomy was performed to expose the olfactory bulb. The olfactory mucosa was electrically stimulated using a concentric circular needle electrode (200 μm in diameter) that delivered a constant current stimulus of 0.5 mA, 0.3 ms, at a rate of 1 Hz. Field potentials were recorded from a region of the ipsilateral olfactory bulb 1000 μm lateral to the midline, 6000 μm rostral to the bregma, and 1000 μm ventral to the surface. The recording microelectrodes used in this experiment were glass capillary pipettes filled with 0.5 M KCl with a tip resistance of 3–6 MΩ. To improve the signal-to-noise ratio of recordings, the olfactory mucosa stimulation-induced responses were averaged for 32 stimulus trials. The magnitude of field potential was quantified by measuring the time integral of the evoked field potential.

### Statistical analysis

All numerical data obtained are expressed as means ± standard error of the mean (SEM). For statistical analysis of the data, the Mann–Whitney *U* test was used to determine differences in average values between two groups. For three groups, the two-way ANOVA was used, and post hoc comparisons were performed by the Fisher’s PLSD method for a gene expression study and by the Bonferroni’s method for analyses of a histological study. The chi-square (*χ*^2^) test for independence was used to test for differences in ratio. Differences were regarded as significant when *p* < 0.05 for two group and *p* < 0.016 for three group comparisons.

## Results

### HMGB1 expression

**Fig. 2:**
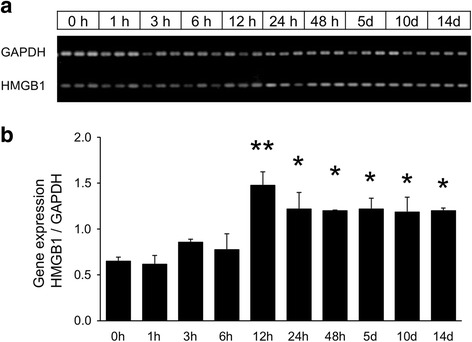
HMGB1 gene expression. **a** PCR data for GAPDH and HMGB1 present in the olfactory bulb 0 h to 14 days after NTx injury. **b** Results of semi-quantitative PCR measurements. Ratio of HMGB1 genes at 1-h–14-day time points after NTx injury are significantly increased compared to the 0-h time point. **p < 0.0001, *p < 0.005

**Fig. 3:**
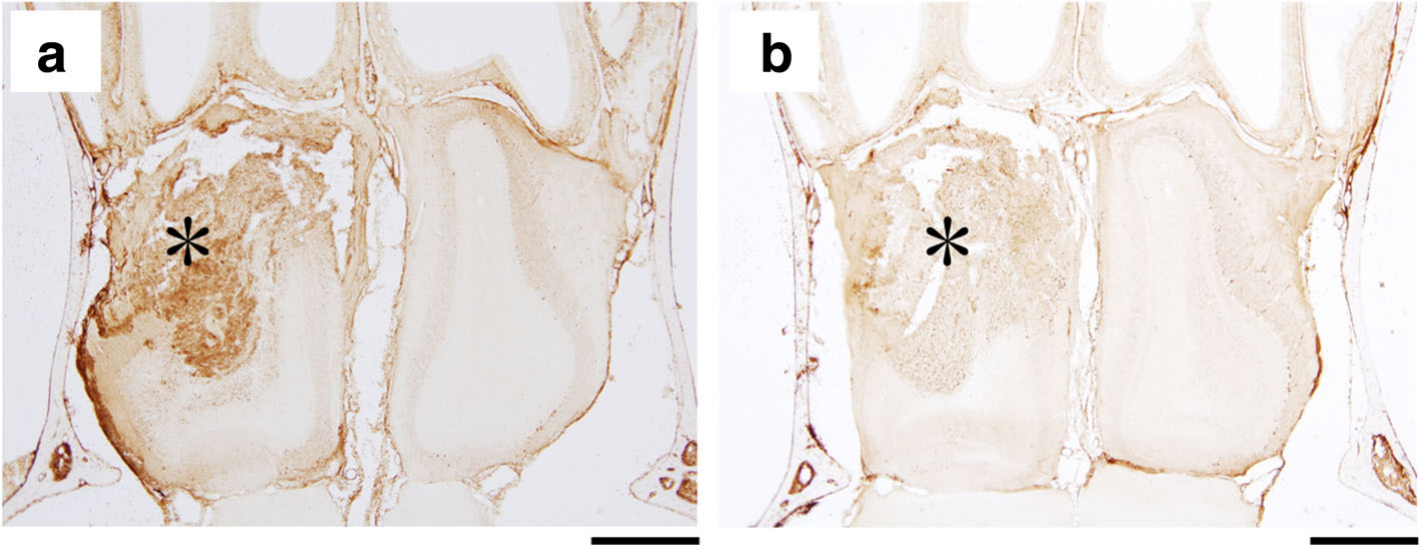
HMGB1 protein localization after NTx injury. Histological sections at 12 (**a**) and 24 h (**b**) after left NTx injury. HMGB1 protein is localized in and around injury-associated tissue and anterior part of the olfactory bulb (*) of the NTx side. Calibration bar = 500 μm

A semi-quantitative PCR shows that there are significant increases in HMGB1 gene expression 12 h after the NTx or later, with a peak 12 h after the NTx injury before beginning to decrease (Fig. 2). Immunohistochemical staining using anti-HMGB1 antibody showed strong positive reaction in the injury-associated tissue and damaged anterior part of the olfactory bulb of the NTx side compared to the control side (Fig. 3). These results showed that HMGB1, the target of its antibody, increased at damaged olfactory tissues after the NTx.

### Effects of anti-HMGB1 antibody injection

**Fig. 4:**
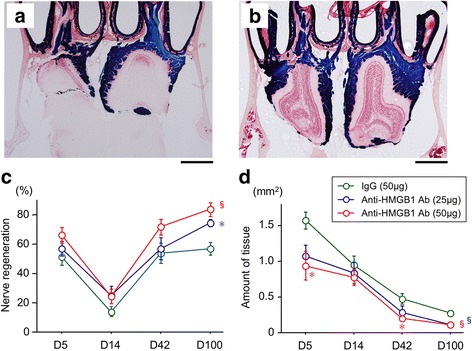
Effects of anti-HMGB1 antibody on recovery from olfactory NTx injury. Histological sections illustrating control IgG (50 μg, **a**)- and anti-HMGB1 antibody (50 μg, **b**)-injected mice at 100 days after NTx injury. Calibration bar = 500 μm. Quantitative measurements showing the time course and comparison of changes in the amount of injury-associated tissue (**c**) and X-gal stained olfactory nerve innervation to the glomerular layer on the olfactory bulb (**d**) for anti-HMGB1 antibody (low and high doses) and control IgG animals. Asterisk (*) and section sign (§) indicate significant differences (*p < 0.016, §*p* < 0.0005) compared to the control IgG group

To determine if anti-HMGB1 antibody treatment can facilitate recovery of the olfactory nerves after NTx injury, it was injected intraperitoneally in the severe injury model. Figure 4a shows results of the control (IgG) compared to effects of anti-HMGB1 antibody treatment (Fig. 4b). A decrease in the percentage of X-gal (blue) staining on the NTx side at day 5 and day 14 reflects the degeneration of olfactory nerves (Fig. 4c). However, the subsequent increase in blue staining in the nerve and glomerular layers at days 42 and 100 indicate that the regenerating olfactory nerves had reestablished connections with the olfactory bulb. Compared to the IgG controls, a significantly higher level of the nerve recovery was found in the anti-HMGB1 antibody-injected mice at day 100, and this increase was dose-dependent.

Figure 4d shows changes in the amount of injury-associated tissue (glial scar) present on the NTx side. The amounts increased at day 5 and gradually decreased at day 14 and after. The tissue amount in low and high doses of anti-HMGB1 antibody-injected mice was significantly less than that in the control IgG mice.

**Fig. 5:**
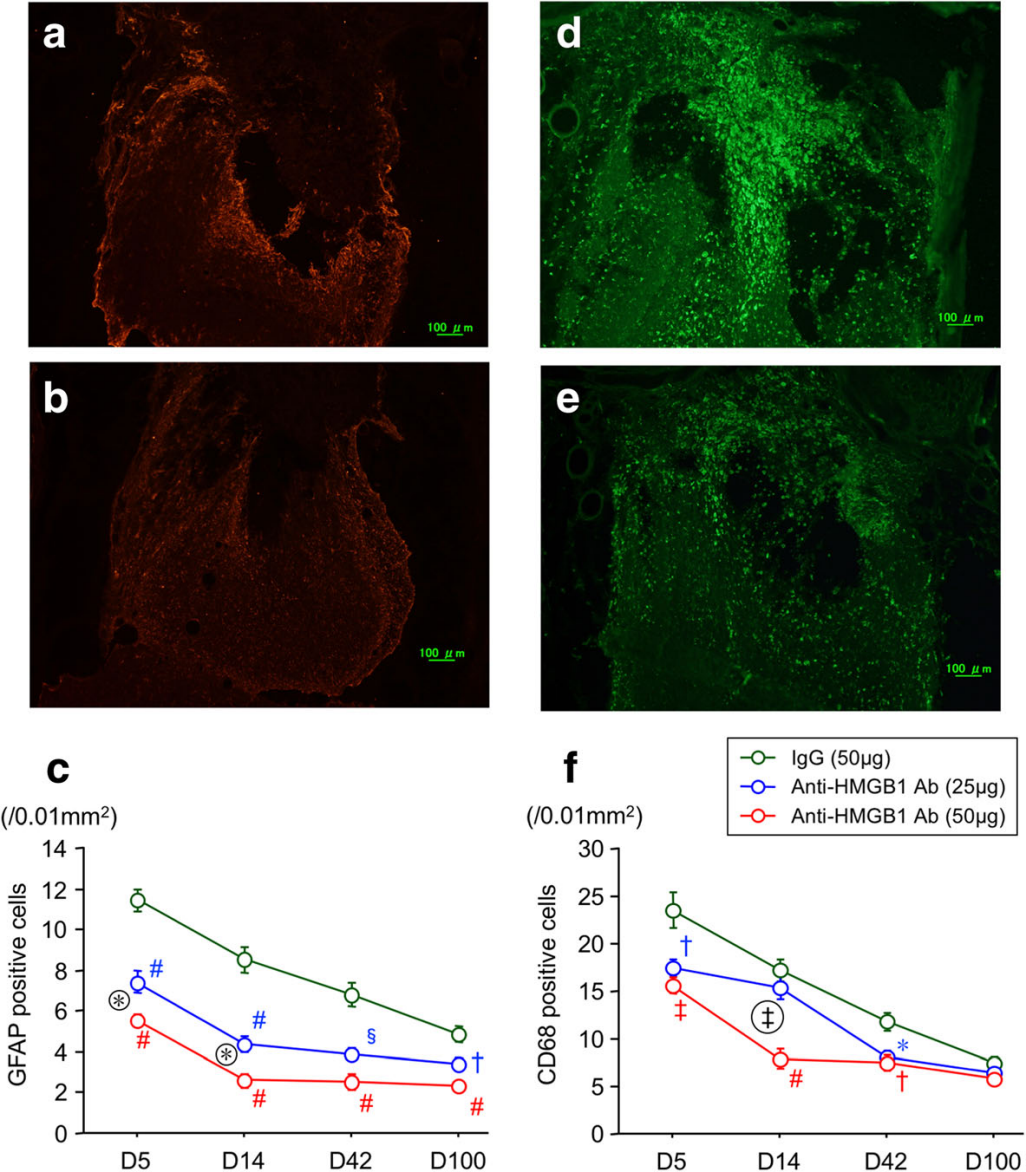
Effects of anti-HMGB1 antibody on glial and inflammatory cells after NTx injury. Histological sections illustrating the left side (NTx side) of the olfactory bulbs applied GFAP (**a**, **b**) and CD68 (**d**, **e**) immunohistochemical staining for control IgG (**a**, **d**) and high doses of anti-HMGB1 antibody (50 μg, **b**, **e**)-injected animals 5 days after the NTx injury. Quantitative measurements showing the time course and comparison of changes in the amount of GFAP-positive cells (**c**) and CD68-positive cells (**f**). Data plotted are means ± SE. Significant differences are shown as *p < 0.016, †*p* < 0.005, §*p* < 0.0005, and #*p* < 0.0001 compared to the control IgG group. Asterisk (*) and double dagger (‡) in circles indicate significant differences (*p < 0.016, ‡*p* < 0.001) between the low- and high-dose anti-HMGB1 antibody groups

Both GFAP-positive cells and CD68-positive cells increased on the NTx side in the olfactory bulbs at day 5 and gradually decreased at day 14 and later recovery times (Fig. 5). With anti-HMGB1 antibody treatment, the number of both GFAP and CD68 cells decreased compared with those in control IgG mice in a dose-dependent manner.

### Olfactory function tests

An olfactory function test using avoidance conditioning behavior to cycloheximide was administered to mice injected with anti-HMGB1 antibody (50 μg in 0.5 ml saline) and control IgG (50 μg in 0.5 ml saline) before and after the NTx. For the anti-HMGB1 antibody group, 11 of 16 (69%) mice achieved a score of 100% on the olfactory function test after NTx, indicating that their olfactory function had recovered (Table 1). The average time required for behavioral recovery in the 11 mice was 48 ± 7 days. For the control saline group, however, only 3 of 15 (20%) mice recovered their olfactory function (98, 77, and 35 days, 70 ± 19 days). Anti-HMGB1 antibody-injected mice showed a significantly higher rate of olfactory function recovery than control IgG mice (*p* < 0.05).

**Table 1:** Results of the olfactory function test

	IgG	Anti-HMGB1 antibody	
Recovering/total mice (*n*/*n*)	3/15	11/16	
% of recovering mice	20%	69%	*p* < 0.05
Days needed for recovery	70 ± 19	48 ± 7	N.S.

### Electric field potential in olfactory bulbs evoked by olfactory mucosa stimulation

**Fig. 6:**
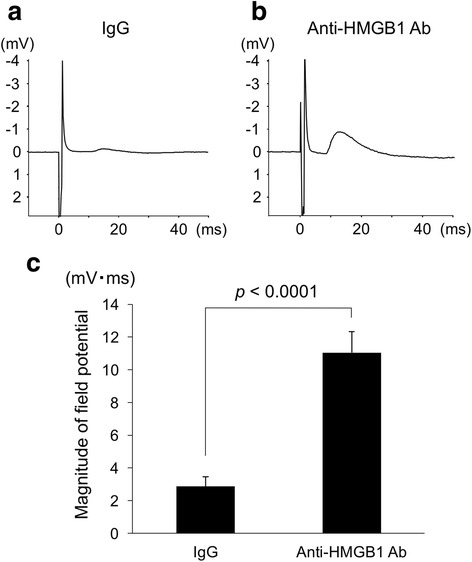
Electric field potential in olfactory bulbs evoked by olfactory mucosa stimulation. Evoked field potential from control IgG mouse that did not show any olfactory behavioral function recovery (**a**), and an anti-HMGB1 antibody mouse that showed olfactory function recovery (**b**) in the olfactory functional test at 100 days after the NTx injury. **c** Comparison of the time integral magnitude of field potentials, comparing the anti-HMGB1 antibody (50 μg, *n* = 16) and control IgG (*n* = 15) groups

Positive field potentials induced by electrical stimulation of olfactory mucosa were successfully recorded from the mice that showed functional recovery in the olfactory test while little or no field potentials were observed in mice that did not recover olfaction function (Fig. 6). The anti-HMGB1 antibody group (*n* = 16) showed significantly larger field potentials than the IgG control group (*n* = 15).

## Discussion

The present study shows that blockade of HMGB1 can suppress local infiltration of inflammatory cells and glial scar tissue formation and subsequently facilitate morphological and functional recovery of the olfactory system. These results coincide with our previous reports that demonstrated olfactory nerve regeneration by systemic administration of steroids, anti-IL-6 receptor antibody, and TNF-α blocker, all of which suppress local inflammation and glial scar formation in the mouse injury model [12–14]. Results of the previous and present studies are consistent although the anti-HMGB1 antibody is pharmacologically different from those agents, which means that local inflammation and gliosis can suppress olfactory nerve regeneration and recovery and that nonspecific anti-inflammatory treatment can be useful for olfactory system recovery after nerve injury.

HMGB1 released from the nucleus of damaged or necrotic cell activates macrophage/microglia via RAGE, TLR2, TLR4, and macrophage-1 antigen (Mac1) [21, 29]. The activated cell mediates neuroinflammation by secreting nitrite and cytokines as TNF-α, IL-1β, IL-6, and IL-8, thereby inducing necrosis of other neurons and resulting in further HMGB1 release. Thus, there is a positive feedback circuit between HMGB1 and proinflammatory cytokines. Neuroinflammation is associated with reactive astrocytes, which induce gliosis and scar formation and enhance release of inflammatory cytokines, leading to limitation of axonal regeneration [30]. A previous study reported that TLR2 and TLR4 are expressed on astrocytes and anti-HMGB1 antibody decreased GFAP expression, suggesting that HMGB1 may activate astrocytes though these receptors [31]. These results are consistent with our present results showing that proliferations of GFAP-positive reactive astrocytes and CD68-positive macrophage/microglia observed after NTx were suppressed in anti-HMGB1 antibody-injected animals.

The process of neural injury consists of two phases, the primary injury and the secondary injury [32]. Within a day after the primary injury caused by direct mechanical damage to a neuronal tissue, proinflammatory cytokines are produced and released at the lesion site in the second phase. These cytokines induce expansion of tissue damage by an increase in vascular permeability, massive recruitment of inflammatory cells such as neutrophils and macrophages/microglia, which produce proteolytic enzymes and reactive oxygen species causing myelin degeneration and neuronal apoptosis. These reactions lead to cell death and scar formation in the nervous system, resulting in neurological deficits. A previous study reported that serum TNF-α, one of the proinflammatory cytokines, significantly increases within 1–2 h, followed by a significant increase in serum HMGB1 at 16–32 h after lipopolysaccharide (LPS) stimulation [33]. This is consistent with our previous and present PCR results that TNF-α gene significantly expressed at an hour and HMGB1 gene significantly expressed at 12 h after the olfactory NTx [14]. Therefore, the amount of HMGB1 is boosted by secreted TNF-α in the secondary injury while a mechanical damage in the primary injury immediately releases a triggering HMGB1 that induces TNF-α from the macrophage/microglia. Considering these multiple implications of HMGB1 in neuroinflammation after injury, the anti-inflammatory strategy using anti-HMGB1 can be regarded as an advantageous and ideal treatment for olfactory dysfunction in an acute phase of head trauma.

Another advantage to using anti-HMGB1 antibody is that its clinical safety is considerably promising. There have been no or few clinical and experimental reports showing side effects due to in vivo administration of anti-HMGB1 antibody, compared with steroids, which are not recommended for patients with head injury because of no significant effects on morbidity and mortality and concerns about their side effects. Anti-HMGB1 antibody has been shown to be useful for prevention of brain edema, ischemic brain injury, and delayed cerebral vasospasm after subarachnoid hemorrhage [25, 34, 35]. It is also useful for prevention of septic infection [36, 37], while anti-IL-6 antibody and TNF-α blocker sometimes aggravate infectious diseases [18, 19]. Since anti-HMGB1 antibody is efficacious against other brain damages occurring together with olfactory dysfunction, it can be a preferable agent to use in severe head injury cases.

Future work is needed to find optimal timing and dose for drug administration. Our results show that the timing used in this study is appropriate for good recovery of the olfactory system. In many clinical head injury cases, however, olfactory dysfunction is not diagnosed until weeks or months after the injury, since patients and medical staff usually direct their attention to more critical life-threatening injuries and often overlook any olfactory impairment. Therefore, future studies are needed to determine effective administration timing and dose for treatment using anti-HMGB1 antibody during chronic olfactory dysfunction that is noted at later stages after head trauma, as well as efficacious combination with other drugs having synergistic effects.

## Conclusions

The present study revealed that inactivation of HMGB1 using its antibody in the acute phase of olfactory nerve injury can contribute to facilitate functional recovery of the olfactory system by suppressing local infiltration of inflammatory cells and glial scar tissue formation. This may provide a new therapeutic strategy for the treatment of olfactory dysfunction following head injuries safely without severe drug-induced side effects.

## Abbreviations

CD68: Cluster of differentiation 68
EDTA: Ethylenediaminetetraacetic acid
EGTA: Ethylene glycol tetraacetic acid
GFAP: Glial fibrillary acidic protein
HMGB1: High mobility group box 1
IL: Interleukin
Mac1: Macrophage-1 antigen
NIH: National Institute of Health
NTx: Olfactory nerve transection
OMP: Olfactory marker protein
PCR: Polymerase chain reaction
RAGE: Receptor for advanced glycation end product
TLR: Toll-like receptor
TNF: Tumor necrosis factor
*χ*^2^: Chi-square

## References

[1] Miwa T, Furukawa M, Tsukatani T, Costanzo RM, DiNardo LJ, Reiter ER (2001). Impact of olfactory impairment on quality of life and disability. Arch Otolaryngol Head Neck Surg.

[2] Santos DV, Reiter ER, DiNardo LJ, Costanzo RM (2004). Hazardous events associated with impaired olfactory function. Arch Otolaryngol Head Neck Surg.

[3] Costanzo RM, Reiter ER, Yelverton JC, Zasler ND, Katz DI, Zafonte RD (2012). Smell and taste. Brain injury medicine: principles and practice.

[4] Sumner D (1964). Post-traumatic anosmia. Brain.

[5] Zusho H (1982). Posttraumatic anosmia. Arch Otolaryngol.

[6] Costanzo RM, Becker DP, Meiselman HL, Rivlin RS (1986). Smell and taste disorders in head injury and neurosurgery patients. Clinical measurements of taste and smell.

[7] Jimenez DF, Sundrani S, Barone CM (1997). Posttraumatic anosmia in craniofacial trauma. J Craniomaxillofac Trauma.

[8] London B, Nabet B, Fisher AR, White B, Sammel MD, Doty RL (2008). Predictors of prognosis in patients with olfactory disturbance. Ann Neurol.

[9] Delank KW, Stoll W (1998). Olfactory function after functional endoscopic sinus surgery for chronic sinusitis. Rhinology.

[10] Kobayashi M, Imanishi Y, Ishikawa M, Nishida K, Adachi M, Oishi M, Nakamura S, Sakaida H, Majima Y (2005). Safety and usefulness of the long-term intranasal topical treatment with steroids for olfactory dysfunction. Nippon Jibiinkoka Gakkai Kaiho.

[11] Miwa T, Uramoto N, Tsukatani T, Furukawa M (2005). Middle turbinate fenestration method: a new technique for the treatment of olfactory disturbance due to chronic sinusitis. Chem Senses.

[12] Kobayashi M, Costanzo RM (2009). Olfactory nerve recovery following mild and severe injury and the efficacy of dexamethasone treatment. Chem Senses.

[13] Kobayashi M, Tamari K, Miyamura T, Takeuchi K (2013). Blockade of interleukin-6 receptor suppresses inflammatory reaction and facilitates functional recovery following olfactory system injury. Neurosci Res.

[14] Al Salihi MO, Kobayashi M, Tamari K, Miyamura T, Takeuchi K (2017). Tumor necrosis factor-α antagonist suppresses local inflammatory reaction and facilitates olfactory nerve recovery following injury. Auris Nasus Larynx.

[15] Cooper PR, Moody S, Clark WK, Kirkpatrick J, Maravilla K, Gould AL, Drane W (1979). Dexamethasone and severe head injury. A prospective double-blind study. J Neurosurg.

[16] Braakman R, Schouten HJ, Blaauw-van DM, Minderhoud JM (1983). Megadose steroids in severe head injury. Results of a prospective doubleblind clinical trial. J Neurosurg.

[17] Dearden NM, Gibson JS, McDowall DG, Gibson RM, Cameron MM (1986). Effect of high-dose dexamethasone on outcome from severe head injury. J Neurosurg.

[18] Genovese MC, McKay JD, Nasonov EL, Mysler EF, da Silva NA, Alecock E, Woodworth T, Gomez-Reino JJ (2008). Interleukin-6 receptor inhibition with tocilizumab reduces disease activity in rheumatoid arthritis with inadequate response to disease-modifying antirheumatic drugs: the tocilizumab in combination with traditional disease-modifying antirheumatic drug therapy study. Arthritis Rheum.

[19] Dogra S, Khullar G (2013). Tumor necrosis factor-α antagonists: side effects and their management. Indian J Dermatol Venereol Leprol.

[20] Lotze MT, Tracey KJ (2005). High-mobility group box 1 protein (HMGB1): nuclear weapon in the immune arsenal. Nat Rev Immunol.

[21] Muhammad S, Barakat W, Stoyanov S, Murikinati S, Yang H, Tracey KJ, Bendszus M, Rossetti G, Nawroth PP, Bierhaus A, Schwaninger M (2008). The HMGB1 receptor RAGE mediates ischemic brain damage. J Neurosci.

[22] Maroso M, Balosso S, Ravizza T, Liu J, Aronica E, Iyer AM, Rossetti C, Molteni M, Casalgrandi M, Manfredi AA, Bianchi ME, Vezzani A (2010). Toll-like receptor 4 and high-mobility group box-1 are involved in ictogenesis and can be targeted to reduce seizures. Nat Med.

[23] Kawabata H, Setoguchi T, Yone K, Souda M, Yoshida H, Kawahara K, Maruyama I, Komiya S (2010). High mobility group box 1 is upregulated after spinal cord injury and is associated with neuronal cell apoptosis. Spine.

[24] Kikuchi K, Uchikado H, Miura N, Morimoto Y, Ito T, Tancharoen S, Miyata K, Sakamoto R, Kikuchi C, Iida N, Shiomi N, Kuramoto T, Miyagi N, Kawahara KI (2011). HMGB1 as a therapeutic target in spinal cord injury: a hypothesis for novel therapy development. Exp Ther Med.

[25] Okuma Y, Liu K, Wake H, Zhang J, Maruo T, Date I, Yoshino T, Ohtsuka A, Otani N, Tomura S, Shima K, Yamamoto Y, Yamamoto H, Takahashi HK, Mori S, Nishibori M (2012). Anti-high mobility group box-1 antibody therapy for traumatic brain injury. Ann Neurol.

[26] Mombaerts P, Wang F, Dulac C (1996). Visualizing an olfactory sensory map. Cell.

[27] Farbman AI, Margolis FL (1980). Olfactory marker protein during ontogeny: immunohistochemical localization. Dev Biol.

[28] Silver J, Miller JH (2004). Regeneration beyond the glial scar. Nat Rev Neurosci.

[29] Fang P, Schachner M, Shen YQ (2012). HMGB1 in development and diseases of the central nervous system. Mol Neurobiol.

[30] Sofroniew MV (2005). Reactive astrocytes in neural repair and protection. Neuroscientist.

[31] Ren PC, Zhang Y, Zhang XD, An LJ, Lv HG, He J, Gao CJ, Sun XD (2012). High-mobility group box 1 contributes to mechanical allodynia and spinal astrocytic activation in a mouse model of type 2 diabetes. Brain Res Bull.

[32] Esposito E, Cuzzocrea S (2009). TNF-alpha as a therapeutic target in inflammatory diseases, ischemia-reperfusion injury and trauma. Curr Med Chem.

[33] Wang H, Yang H, Czura CJ, Sama AE, Tracey KJ (2001). HMGB1 as a late mediator of lethal systemic inflammation. Am J Respir Crit Care Med.

[34] Xu M, Zhou GM, Wang LH, Zhu L, Liu JM, Wang XD, Li HT, Chen L (2016). Inhibiting high-mobility group box 1 (HMGB1) attenuates inflammatory cytokine expression and neurological deficit in ischemic brain injury following cardiac arrest in rats. Inflammation.

[35] Haruma J, Teshigawara K, Hishikawa T, Wang D, Liu K, Wake H, Mori S, Takahashi HK, Sugiu K, Date I, Nishibori M (2016). Anti-high mobility group box-1 (HMGB1) antibody attenuates delayed cerebral vasospasm and brain injury after subarachnoid hemorrhage in rats. Sci Rep.

[36] Wang H, Li W, Goldstein R, Tracey KJ, Sama AE (2007). HMGB1 as a potential therapeutic target. Novartis Found Symp.

[37] Wang H, Yang H, Tracey KJ (2004). Extracellular role of HMGB1 in inflammation and sepsis. J Intern Med.

